# Isolated anti-Ku antibody in scleroderma-myositis overlap syndrome: the histo-pathological patern

**DOI:** 10.1186/1479-5876-10-S3-P57

**Published:** 2012-11-28

**Authors:** Bernard Azanmene, Valerie Badot, Severine Verlinden, Maria Josee Fernandez-Lopez, Hazim Kadhim, Francis Corazza, Wolfram Fink, Anne Peretz, Jacques Bentin

**Affiliations:** 1 Dept. of Rheumatology, Brugmann University Hospital (U.L.B.), Brussels, Belgium; 2 Dept. of Pathology, Brugmann University Hospital (U.L.B.), Brussels, Belgium; 3 Dept. of Clinical Biology, Brugmann University Hospital (U.L.B.), Brussels, Belgium; 4 Dept. of Dermatology, Brugmann University Hospital (U.L.B.), Brussels, Belgium

## 

An 18-years old African girl, suffered for almost 4 years of polyarthralgia, joint contractures and a hardcover appearance of the skin suggestive of scleroderma. The clinical picture and a high level of CPK suggested Polymyositis (PM).

Search for anti nuclear Antibody (ANA) showed speckled pattern at 1/2500 titre, and subscreening revealed isolated anti-Ku antibody. This was more often reported in association with PM - SSc overlap syndrome.

Skin biopsy favored linear scleroderma.

Interestingly, muscle biopsy showed features typical of dermatomyositis (DM).

No endocrine disorder or underlying mitotic process was registered.

Corticosteroid therapy was initiated followed by maintenance therapy with methotrexate; the outcome was favorable.

This case illustrates the interest of anti-Ku screening in the diagnostic work-up. Our observation thus emphasizes the possible occurrence of isolated anti-Ku antibody expression in an overlap syndrome comprising SSc-DM association. Such association/linkage (implicating DM rather than PM) is, to our knowledge, very rarely well reported. Moreover, the therapeutic response seems favorable in such condition.

We found one biopsy-confirmed observation of SSc-Inflammatory myositis (IM) overlap syndrome in an anti-Ku positive patient [1,2]. This later case was initially reported as SSc-PM overlap syndrome. Interestingly, follow-up showed evolution towards typical features of DM, and hence an overlap syndrome of SSc-DM association.

Thus, our observation highlights the necessity/interest of biopsy to ascertain the precise nature of myositis in an overlap syndrome associating SSc and Myositis Clinico-pathological criteria in inflammatory myositis have been reviewed by *Chérin P* et al., [3].

Anti-ku Ab is knowingly reported in a context of myositis. Thus our observation and review of the literature suggest that in the presence of isolated anti-Ku Ab in a SSc-Myositis overlap context, muscle biopsy (that should be done) would tend to show DM (rather than PM) in association with the SSc. Such a finding may change the prognosis and the treatment approach of this syndrome. This can only be demonstrated after elaborate muscle biopsy in overlap syndromes, with or without the presence of anti-Ku antibodies.
